# Optical coherence tomography in diagnosing polypoidal choroidal vasculopathy. Looking into the future: a systematic review and meta-analysis

**DOI:** 10.1186/s40942-022-00365-5

**Published:** 2022-02-28

**Authors:** Annisa C. Permadi, Ari Djatikusumo, Gitalisa Andayani Adriono

**Affiliations:** Department of Ophthalmology, Faculty of Medicine Universitas Indonesia – Cipto Mangunkusumo Kirana Eye Hospital, Jakarta, Indonesia

**Keywords:** Polypoidal choroidal vasculopathy, Optical coherence tomography (OCT), Indocyanine green angiography (ICGA), Diagnosis, Meta-analysis

## Abstract

**Background:**

Polypoidal choroidal vasculopathy (PCV) is an exudative maculopathy with features similar to wet age macular degeneration. The incidence of PCV is known to be higher in the Asian population compared to Caucasians. Imaging modality is needed to make the diagnosis of PCV. Although Indocyanine green angiography (ICGA) is still the gold standard, it is not routinely performed in vitreoretinal practice. Thus another imaging modality is currently a popular research area. Spectral domain optical coherence tomography (SD-OCT) has emerged as a new imaging modality mostly available in clinics. Some studies have reported the sensitivity and specificity of SD-OCT in diagnosing PCV with different results and thresholds.

**Methods:**

Relevant studies from PubMed, Science Direct and Google Scholar databases were systematically searched. In random effect models using STATA 14 software, a meta-analysis was performed to determine the pooled diagnostic accuracy. QUADAS 2 was used to evaluate the risk of bias of each study by Revman 5.4 software.

**Results:**

Seven eligible studies which met the inclusion and exclusion criteria were enrolled in this study. A total of 911 eyes were included to investigate the diagnostic accuracy of SD-OCT. As a result, the pooled sensitivity was 0.91 (95% CI 0.87–0.93), specificity 0.88 (95% 0.83–0.92), positive likelihood ratio 8, negative likelihood ratio 11, the area under the summary receiver operating characteristic curve 0.95 (95% CI 0.93–0.97), and diagnostic odds ratio 71.81 (95% CI 38.89–132.74).

**Conclusion:**

SD-OCT provided a high diagnostic value for detecting PCV. Sharply peaked pigment epithelial detachment (PED), notched PED, bubble sign, multiple PED, and double-layer sign were the most common features found in PCV.

## Background

Polypoidal choroidal vasculopathy (PCV) is an exudative maculopathy with features similar to wet age macular degeneration (AMD). It is considered a subtype of AMD characterized by pigment epithelial detachment (PED), retinal detachment and may present with haemorrhage [1]. PCV prevalence in presumed neovascular AMD was 7.8% in the United States, 9.2% in Italian, 8.2% in Greek compared to Asian population such as 23.0–54.7% in Japanese, 22.3–49% in Chinese, and 24.6% in a Korean population [2–4]. In contrast, the incidence of AMD is very high in Caucasians, while both diseases are high in Asians. The average age was reported to be 66 years old in the Chinese population, while Caucasians usually present with PCV at an older age [2, 5]

Clinically, PCV appears as a protruding reddish-orange, spheroid, polyp-like structure around the macula. It is characterized by an inner choroidal vascular network with an aneurysmal bulge that projects outward. Histopathological features indicated arteriosclerosis in the choroidal vessels [6–8]. The vessels exhibited hyalinization and disappearance of choriocapillaris hence massive leakage [8–10]. Histochemistry of PCV showed discontinuity of vascular endothelium, and *smooth muscle actin* (SMA) was negative [6, 11]. This disruption of smooth muscle cells causing dilatation of vessels. Vascular endothelial growth factor (VEGF) antibody was found to be negative in the vascular endothelial cells [6, 12]. This finding revealed the differences between PCV and choroidal neovascularization (CNV); therefore, PCV might not respond to anti-VEGF treatment. Genetic studies have investigated the relationship between PCV and CNV in AMD. They identified many similar genes associated with PCV and CNV, such as *complement factor H* [5–7].

Indocyanine green angiography (ICGA) is the gold standard tool for visualizing the PCV [10, 12, 13]. The higher binding affinity of indocyanine green to plasma proteins prevents it to leak rapidly from choriocapillaris, providing better visualization of a choroidal vessel. Moreover, indocyanine green absorbs and emits near-infrared light, which penetrates RPE, enhancing the view of choroidal lesions [5, 8]. ICGA shows branch vascular network of inner choroidal vessels and aneurysms or dilation at the edge of these vessels giving the appearance of polyps [14, 15]. ICGA is considered a relative safety procedure with anaphylaxis events reported as low as 0.05% [16]. Absolute contraindication of this procedure is in patients with a history of a definite iodine allergy [16]. However, the use of ICGA has become less popular in Optical Coherence Tomography (OCT) era [17, 18]. Not only because OCT is a non-invasive procedure, but it also gives quantitative analysis and saves time [19–21].

OCT is a novel scanning modality that allows cross-sectional images of the retina [19, 22]. It is a non-invasive and quick procedure using infrared light, which is reflected from the reference mirror, and the other is scattered from retina layers [23]. The two reflected beams will produce an interference pattern to obtain an A-Scan. Multiple A-Scan will produce B-Scan which is 2 dimensional image of retina layers. Fourier-domain OCT has two types of OCT, Spectral Domain (SD) OCT and Swept Source (SS) OCT [21, 24]. SS OCT is the latest technology in retinal and choroidal imaging with longer wavelength (1050 nm vs 840 nm in SD OCT) to overcome scattering light by RPE thus providing better visualization from vitreous to choroid. However, with Enhance Depth Imaging (EDI) technique in SD-OCT, it can also be used to visualize the choroid and other structures below RPE in a cross-sectional image [25]. Another advantage provided by SD-OCT is its relatively lower cost compared to SS-OCT, making it is affordable and widely used in most retina clinics [26, 27].

With the proportion of blindness attributable to AMD projected to be increased to 288 million affected people in 2040, it is an urgent need to differentiate the PCV and AMD patients since they have a different approaches in treatment [2, 7]. Differentiation between PCV and wet AMD cannot be made merely on eye examination. As such, imaging modality is crucial to make a sharp diagnosis and the disease evaluation over time. To date, ICGA remains the gold standard tool for diagnosing PCV regardless of its unavailability in many parts of the world [10, 18, 20, 22]. However, the invasive and time-consuming nature of ICGA impedes its practical use for routine treatment follow up. On the other hand, SD-OCT is rapidly evolving as a common tool used by a retina specialists [22]. It provides qualitative and quantitative measurement, quick procedure, lower cost, and non-invasive imaging.

This study was designed to evaluate the overall diagnostic value of OCT compared with ICGA in the detection of PCV by analyzing diagnostic accuracy, including sensitivity, specificity, likelihood ratio, diagnostic ratio and the area under the Summary Receiver Operating Characteristic (SROC) in different studies. SD-OCT characteristic features for diagnosing PCV were aimed as the secondary outcomes.

## Method

### Search strategy and selection criteria

This study was conducted in accordance with Preferred Reporting Items for Systematic Reviews and Meta-analysis (PRISMA) guidelines. Literature searching was conducted using three online databases (PubMed, Science Direct, and Google Scholar) from July 15th to August 10th, 2020. Search terms such as Polypoidal Choroidal Vasculopathy, Indocyanine Green Angiography, Optical Coherence Tomography, diagnosis, or any relevant synonyms were included. There was no limitation in languages and the year of publication. The inclusion criteria were as follows: (1) Studies that reported the analysis of specificity and sensitivity of SD-OCT in detecting PCV, (2) Studies that mentioned the prespecified SD-OCT criteria of PCV, (3) Studies that confirmed the diagnosis using ICGA. The exclusion criteria were as follows: (1) Inaccessible studies, (2) Subjects are not treatment-naïve PCV; including subjects who were followed up after treatment.

### Data extraction

The eligible data was retrieved from each literature that met the criteria. Protocol and included studies were reviewed using software Review Manager (RevMan) V.5.4. The information extracted from each study included the authors, year of study, number of subjects, pre-specified OCT criteria, sensitivity and specificity of OCT were noted. Measured data were analyzed using STATA 14 software. Primary outcomes were sensitivity, specificity, summary ROC, likelihood ratio, and diagnostic odds ratio of OCT in diagnosing PCV. Secondary outcomes were OCT biomarkers and OCT diagnostic criteria. Inconsistency index (I2) test was noted to assess heterogeneity across studies. Pooled sensitivity and sensitivity was measured using a random-effect model since heterogeneity was expected in a meta-analysis of diagnostic accuracy studies.

### Quality assessment

Critical appraisal of each included study was made using QUADAS 2 tool for diagnostic accuracy study. The appraisal tools focused on four domains: patient selection, index test, reference standard, and flow and timing. The study was considered valid if the patient selection based on clinical diagnosis is exudative maculopathy (included PCV or wet AMD), patients received both OCT and ICGA, OCT and ICGA interpretations were assessed independently, diagnosis of PCV by ICGA was made using EVEREST study criteria.

## Results

### Characteristics of the studies identified

**Fig. 1:**
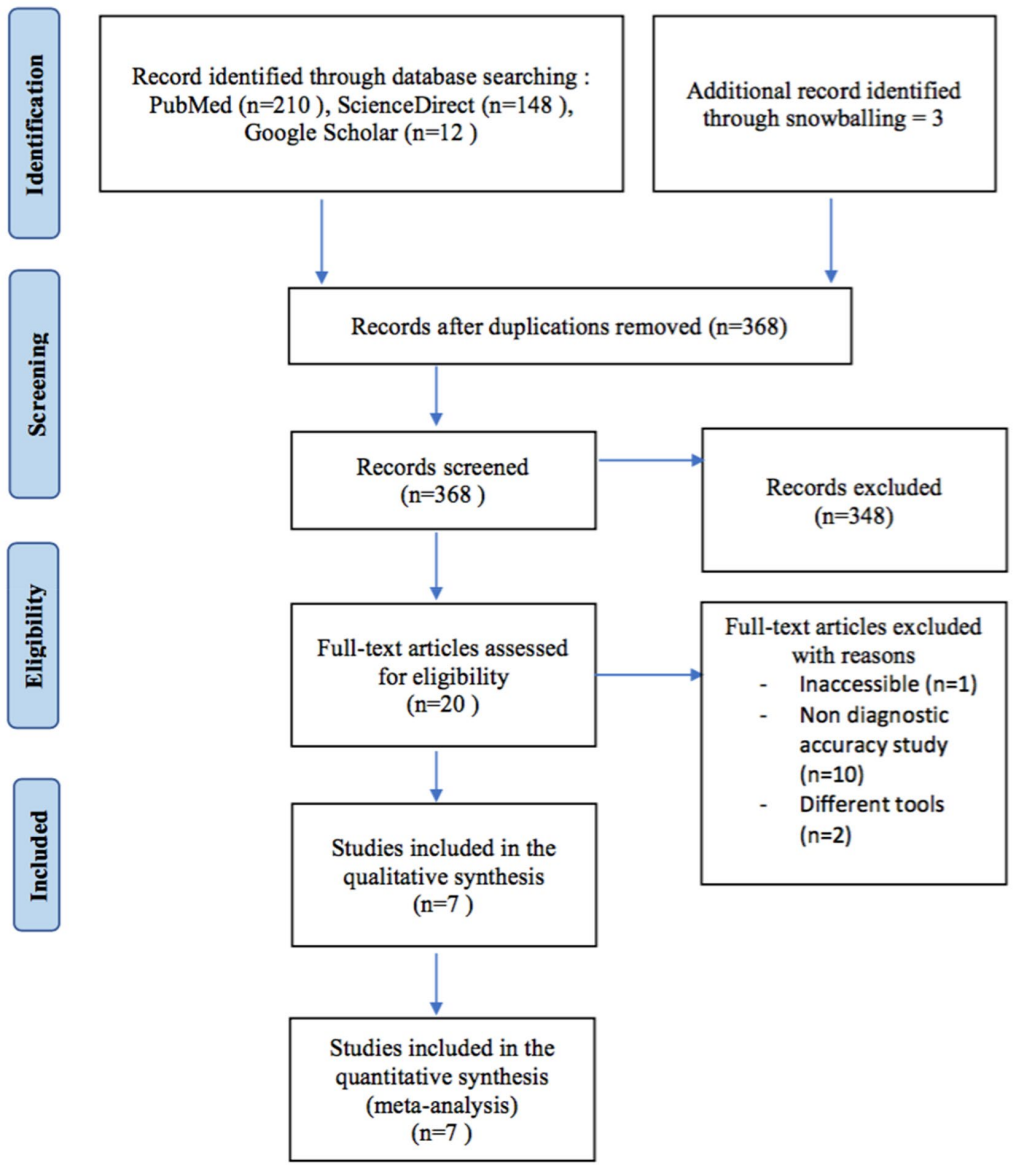
Flowchart of study selection

Our initial search strategy found a total of 368 papers (PubMed: 210, ScienceDirect: 148, Google Scholar: 12). According to inclusion and exclusion criteria, seven studies, including 911 eyes with sufficient data, were selected for the final analysis (Fig. 1). Seven reviewed articles were published from 2014 to 2019, with only one study was a prospective study.

The majority of studies were conducted in Asian region (Thailand, Korea and China) except one study was in United Kingdom (UK). There were no details about race in the study run by De Salvo et al. [20] from the UK. Selected participants in all studies were newly diagnosed exudative maculopathy, including PCV, wet AMD, and chronic serous central chorioretinopathy (CSCR), with only one study excluding CSCR. All included patients received the index test, and reference standard at the same visit except the study by Yang et al. [24]. Only good quality images was included in these studies; however, the indicators were not elaborated. PCV diagnosis was established using EVEREST criteria by ICGA in all studies, whereas prespecified OCT criteria were defined in each study protocol. Two ICGA graders were involved in all studies, with result disagreements were resolved by open adjudications. Four studies determined their least prespecified OCT criteria and were reviewed by 1 to 2 OCT graders. Whereas, studies by Chaikitmongkol et al. [10, 18] and Yang et al. [24] did not set the least criteria, and the images were sent to 3 and 2 OCT grades, respectively. Two-thirds of majority opinions were considered as the final results by Chaikitmongkol et al. [10, 18]. These two studies later analyzed the sensitivity and specificity of every biomarker to make recommended diagnostic criteria.

Each study had similar criteria yet different positive threshold, involving: multiple PED; sharply peaked PED; notched PED; double-layer sign; and the hyperreflective ring surrounding hyporeflective halo underneath PED. Only two studies by Yang et al. [24] and Chang et al. [4] included the choroidal thickness as one of the biomarkers. The only prospective study by Liu et al. [28] created combined biomarkers in one term as Thumb-like polyps, which was defined by any of sharply peaked PED, hyperreflective ring surrounding hyporeflective halo underneath PED and notched PED. Although each study used a different threshold, the sensitivity and specificity appeared to be good with narrow confidence intervals. Table 1 shows the characteristics of 911 eyes from each of the seven studies included in the analysis.

**Table 1:** Characteristic of included studies

No	Study/design	Year	Place	Selected participants	Exclusion criteria	No of eyes/subjects	Graders	OCT criteria	Sensitivity	Specificity
1	De Salvo/retrospective	2014	UK	Serous or hemorrhagic PED	1. Classic exudative AMD 2. Myopic CNV 3. Secondary CNV 4. CSCR 5. Poor quality image 6. Disciform scar	51/44	1 OCT grader ICGA graders Disagreements were resolved by open adjudications	At least 3 of following criteria: 1. Multiple PED 2. Sharpy peaked PED 3. Notched PED 4. Hyperreflective ring surrounding hyporeflective halo underneath PED	0.95 [0.82–0.99]	0.93 [0.66–1.00]
2	Zhang/retrospective	2016	China	Newly diagnosed wet AMD or PCV	1. High myopia 2. Dry AMD 3. Diabetic retinopathy 4. Large-area subretinal hemorrhage	63/62	1 OCT grader 2 ICGA graders. Disagreements were resolved by open adjudications	Two major criteria plus at least 1 minor criteria or at least 3 of minor criteria as follow: Major criteria 1. Sharply peaked PED 2. Double-layer sign Minor criteria 3. Multiple PED 4. Notched PED 5. Hyporeflective halo 6. Intraretinal hard exudate	0.92 [0.74–0.99]	0.89 [0.75–0.97]
3	Chang/retrospective	2016	Korea	Newly diagnosed wet AMD	1. Ocular media opacity 2. End-stage AMD 3. High Myopia 4. Axial length 26.00 mm or greater 5. Immeasurable choroidal thickness 6. Presence of other retinal vascular disorders 7. Subretinal hemorrhage greater than five disc areas in size	263/263	1 OCT grader 2 ICGA graders. Disagreements were resolved by open adjudications	At least three of following criteria: 1. Multiple PED 2. Sharply peaked PED 3. Notched PED 4. Hyperreflective ring surrounding hyporeflective halo underneath PED 5. Intraretinal hard exudate Or at least two of above criteria plus: 1. Subfoveal choroidal thickness 300 nm	0.90 [0.84–0.94]	0.84 [0.77–0.94]
4	Liu/prospective	2016	China	Newly diagnosed wet AMD	1. Secondary choroidal neovascular disease 2. Massive subretinal hemorrhage 3. Large cicatrical lesions 4. Uveitis, diabetic retinopathy, proliferative retinopathy, epiretinal membrane 5. Previous retinal surgery or intraocular injection 6. Ocular media opacity 7. Associated systematic disorders	188/156	2 OCT graders Disagreements were resolved by 3rd experienced grader 2 ICGA graders	At least two of following criteria: 1. PED (single or multiple) 2. Double-layer sign 3.Thumb-like polyps (TLP)	0.89 [0.82–0.94]	0.85 [0.75–0.92]
5	Chaikitmongkol/retrospective	2018	Thailand	Newly diagnosed serous or serosanguinous maculopathy	1. Had previous treatment 2. Coexisting retinal abnormality 2. Ocular media opacity 3. Incomplete imaging (ICGA,OCT)	119/116	3 OCT graders 2 ICGA graders Disagreements were resolved by open adjudications	1. Sharply peaked PED 2. Notched PED 3. Hyperreflective ring surrounding hyporeflective halo underneath PED 4. Double-layer sign Majority rule opinions of 2/3 graders were determined as final result	0.83 [0.70–0.93]	0.83 [0.72–0.91]
6	Chaikitmongkol/retrospective	2019	Thailand	Newly diagnosed serous or serosanguinous maculopathy	1. Had previous treatment 2. Incomplete imaging (ICGA,OCT) 3. Poor quality image	124/120	3 OCT graders 2 ICGA graders. Disagreements were resolved by open adjudications	1. Multiple PED 2. Sharply peaked PED (angle between 70 and 90) 3. Notched or multilobulated PED 4. Hyperreflective ring surrounding hyporeflective halo underneath PED 5. Double-layer sign Majority rule opinions of 2/3 graders were determined as final result	0.95 [0.87–0.99]	0.95 [0.86–0.99]
7	Yang/retrospective	2019	China	Newly diagnosed PCV and wet AMD	1. Other ocular diseases 2. Ocular media opacity 3. Systematic disorders that affect the eyes	103/82	2 OCT graders 2 ICGA graders Disagreements were resolved by open adjudications	1. Multiple PED 2. Sharply peaked PED 3. Notched PED 4. Double-layer sign 5. Bubble sign 6. Pachychoroid 7. Bruch’s membrane depression under serosanguinous PED	0.88 [0.77–0.96]	0.92 [0.81–0.98]

### Quality assessment and publication bias

**Fig. 2:**
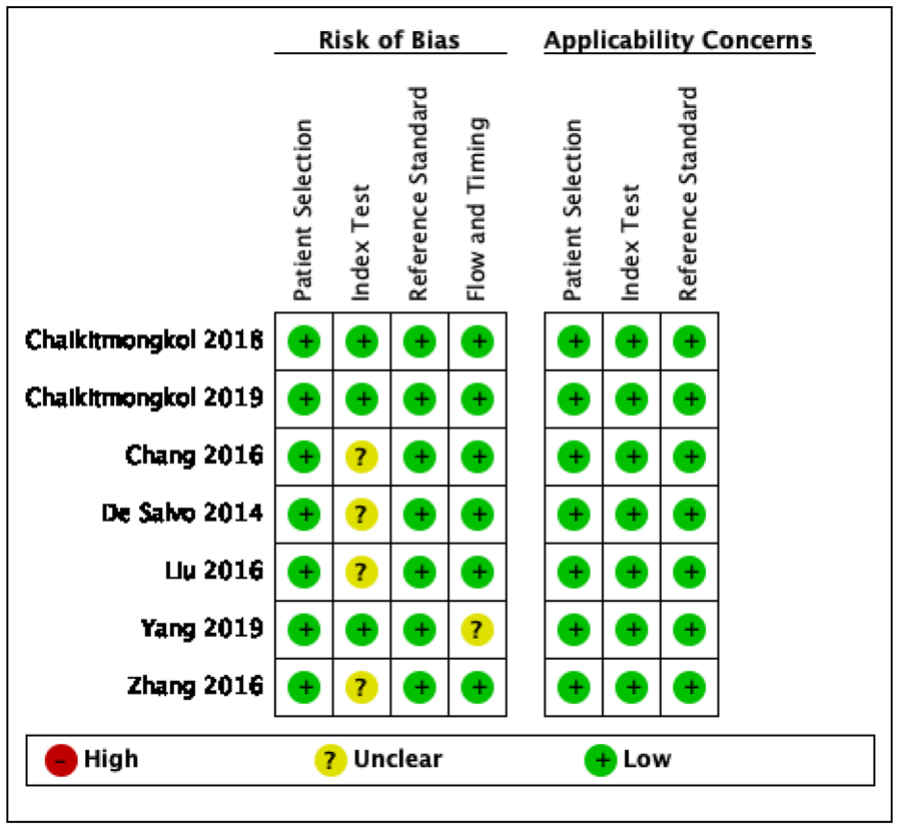
Risk of bias and applicability concerns summary: review authors' judgements about each domain for each included study

**Fig. 3:**
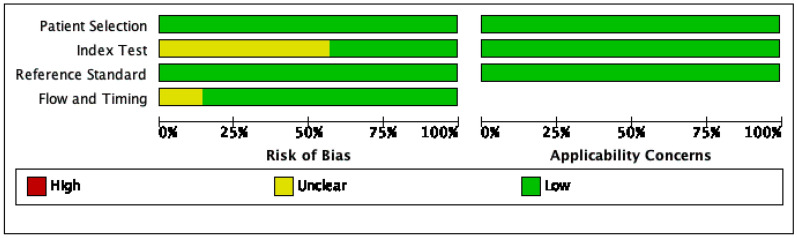
Risk of bias and applicability concerns graph: review authors' judgements about each domain presented as percentages across included studies

All relevant articles were assessed using QUADAS 2 tool diagnostic accuracy study. Noted overall studies have a low risk of bias. Index test interpretation in De Salvo et al. [20], Zhang et al. [13], Chang et al s was made by one grader, in addition, same graders for OCT and ICGA in Liu et al. [28] that may lead to potential information bias. Yang et al. [24] performed ICGA and OCT on a different day that may cause condition bias. (Fig. 2) Overall bias assessment identified low-risk bias all in all studies (Fig. 3).

### Diagnostic performance and clinical value

**Fig. 4:**
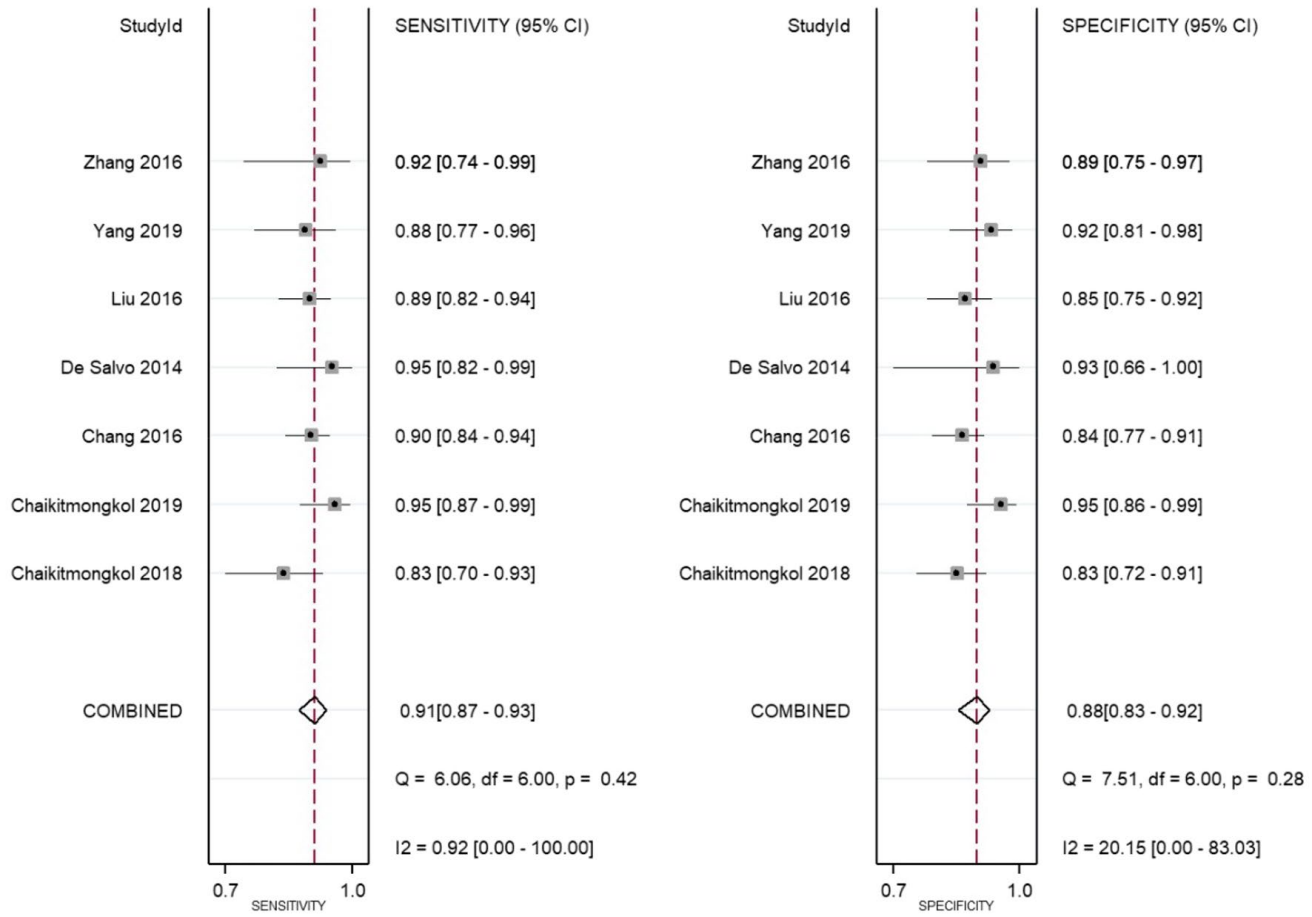
Forest plot for sensitivity and specificity showed excellent result for OCT alone in diagnosing PCV, despite the different threshold used in each study. Note that the heterogeneity was low, indicating a high certainty of evidence

**Fig. 5:**
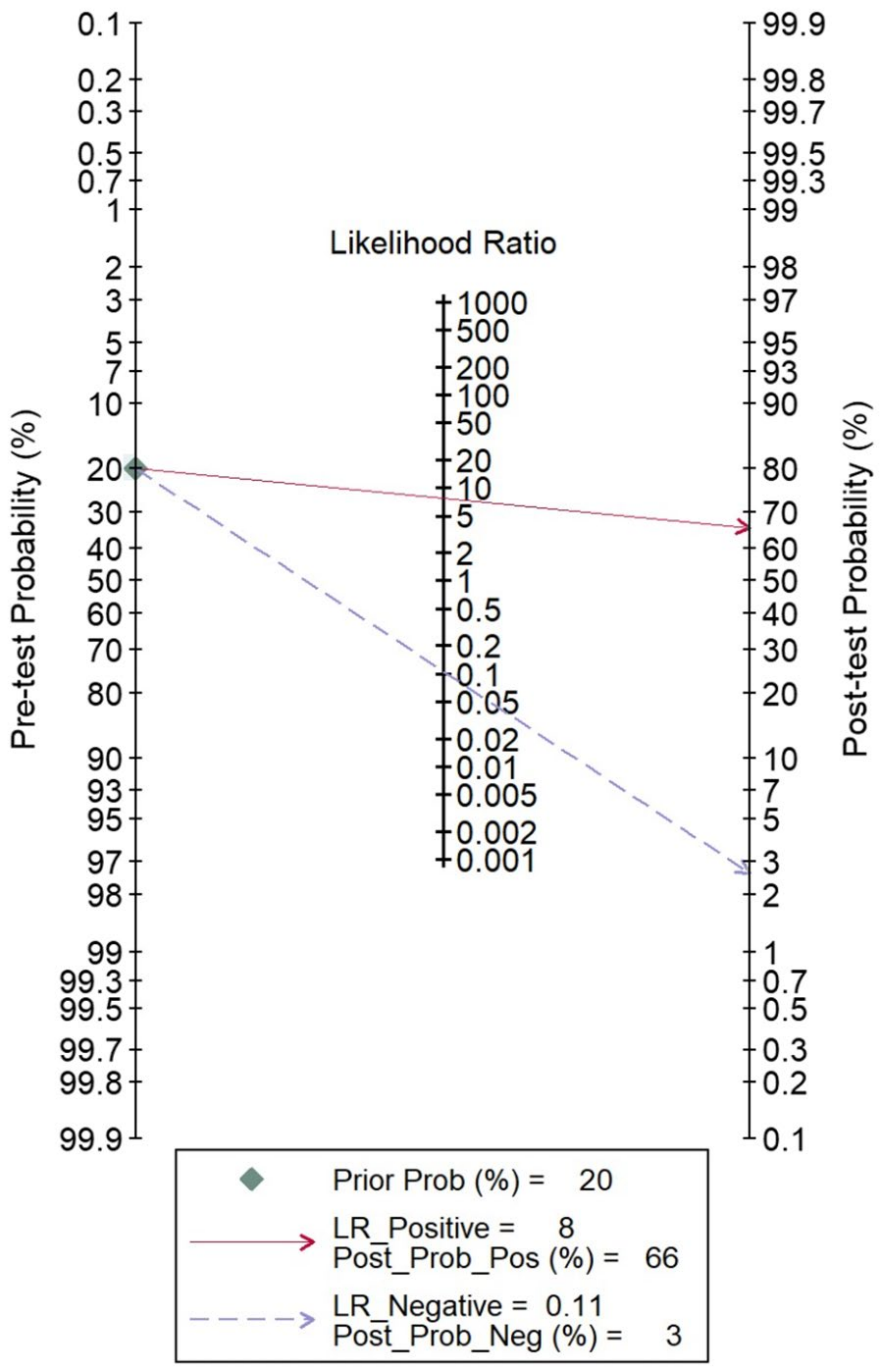
Fagan’s nomogram displayed the value of positive LR at 8 and negative LR at 0.11 specified that OCT performed well at distinguishing PCV and non-PCV. Increase of pre-test to post-test probability way suggested to confirm the diagnosis

Pooled sensitivity and specificity of OCT using random-effect model had excellent values up to 0.91 (0.87–0.93) and 0.88 (0.83–0.92), respectively (Fig. 4) It implied that OCT could detect 91% of patients with PCV and rule out about 88% of a patient without PCV. Inconsistency index (I2) was 0.92 for sensitivity and 20.15 for specificity indicated low heterogeneity across the study. Moreover, positive and negative likelihood ratios (LR) showed remarkable results, with positive LR at eight and negative LR at 0.11 (Fig. 5) This signified that patients with PCV would be more likely to have positive results eight times compared to patients without the disease. In contrast, there is a 0.11% chance that patients with PCV will be tested negative by OCT. Pre-test and post-test probability, as demonstrated in Fagan’s nomogram, escalated from 0.20 to 0.66, which may guarantee initiation of treatment (Fig. 5). All these parameters revealed that OCT performed a great diagnostic tool for detecting PCV.

**Fig. 6:**
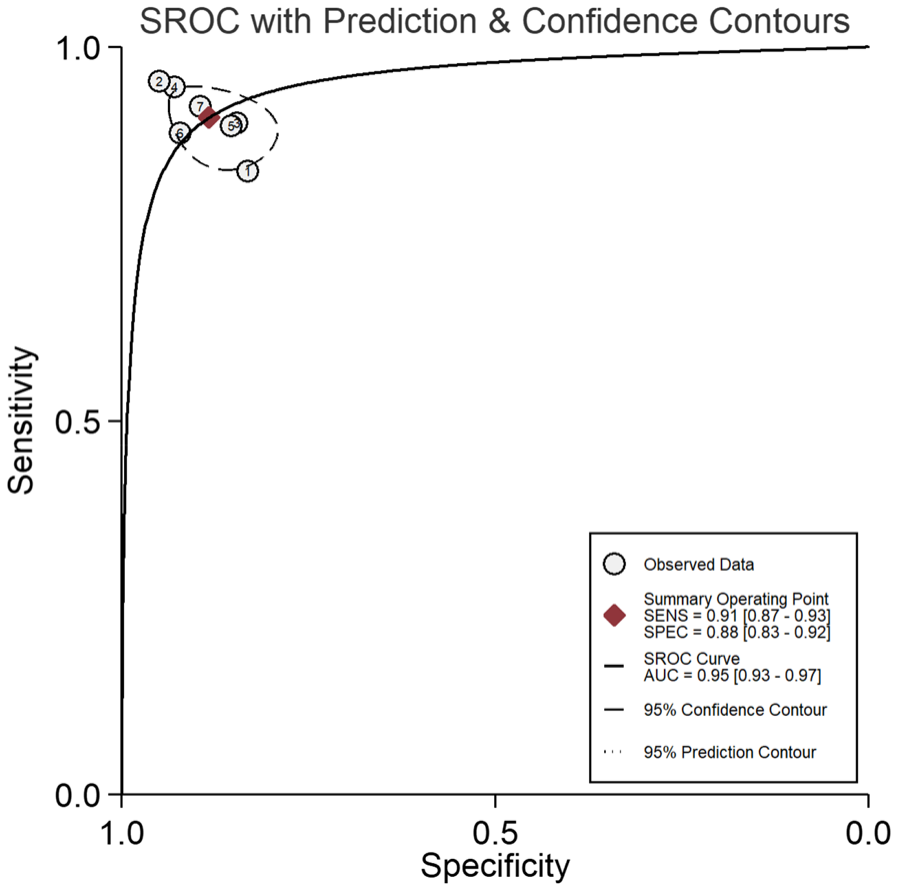
Bivariate SROC showed high AUC at 0.95 which is considered excellent. This area measured discrimination, that was, the ability of OCT to correctly classify those with and without the disease

SROC using a bivariate model depicted the relationship between-test sensitivity and specificity across a study with an AUC value of 0.95 (0.93–0.97). This result was considered excellent as it told how much OCT is capable of distinguishing PCV and not PCV. This graph also showed the expected trade-off in sensitivity and specificity, although the positivity threshold across studies varied. The 95% prediction contour demonstrated the true sensitivity and specificity of a future study should lie despite the extent of statistical heterogeneity (Fig. 6).

**Fig. 7:**
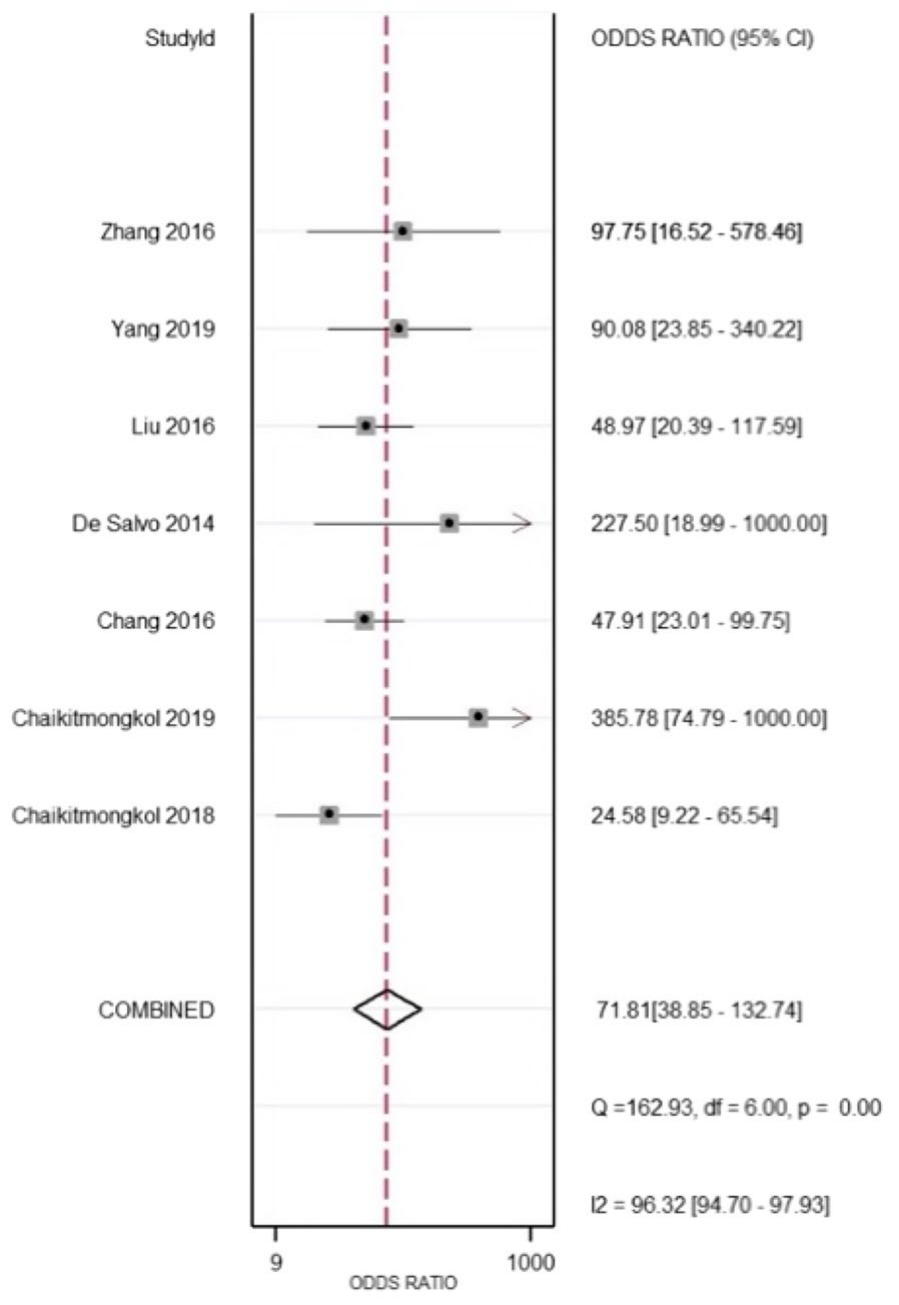
The diagnostic odds ratio (DOR) is the ratio of positive likelihood ratio compared to negative likelihood ratio. It is the odds that the test produces positive results compared to the odds of negative results. DOR of 71.81 (38.89–132.74) revealed a good test performance of OCT

The diagnostic odds ratio (DOR) also gave us strong results about how the odds of OCT is obtaining a positive result in a patient with PCV rather than without PCV. DOR of 71.81 (38.89–132.74) reflected that OCT had excellent discriminatory power regardless of different positive thresholds (Fig. 7).

Similar OCT biomarkers for PCV were noted in each study. Although the threshold for positive results varied, the sensitivity and specificity for different thresholds used by each study remained good. Only five studies showed the value of every biomarker in diagnosing PCV. Nonetheless, the value was described differently using comparison analysis between PCV and non PCV or in sensitivity, specificity and AUC. It was shown that multiple PED, sharply peaked PED, notched PED, hyperreflective ring surrounding hyporeflective halo underneath PED, and double-layer sign were distinguishing features of PCV to other diseases such as wet AMD. Only two studies included choroidal thickness as the biomarker. Other different criteria were used by Yang et al. [22], which mentioned Bruch’s membrane depression under serosanguinous PED.

De Salvo et al. [20] showed all four biomarkers such as multiple PED, sharply peaked PED, notched PED, and the hyperreflective ring surrounding hyporeflective halo underneath PED were statistically significant to be found in PCV rather than wet AMD. Zhang et al. [13] defined the criteria as strategies a and b, which had different least criteria to analyze the sensitivity of specificity. Strategy b showed higher sensitivity and specificity, which sharply peaked PED and double-layer sign must be found in OCT, or other three features of multiple PED notched PED, hyporeflective halo and hard exudate in the retina.

Studies run by Chang et al. [4] and Yang et al. [24] proposed choroidal thickness as an OCT biomarker of PCV. Subfoveal choroidal thickness 300 nm or more was stated as diagnostic criteria in Chang et al. [4], whereas Yang et al. [24] only mentioned pachychoroid. Chaikitmongkol et al. [18] found that notched and multilobulated PED had the highest sensitivity, specificity and AUC value amongst other biomarkers, followed by sharply peaked PED and the hyperreflective ring surrounding hyporeflective halo underneath PED. These three biomarkers were recommended as major criteria by Chaikitmongkol et al. [18] in diagnosing PCV in addition to hemorrhagic PED in fundus examination. It showed that 2 or more of 4 major criteria highly suggested PCV lesion.

Another study by Yang et al. [24] made criteria recommendations after testing the sensitivity, specificity and AUC of given biomarkers. Sharply peaked PED, notched PED, bubble sign (hyperreflective ring surrounding hyporeflective halo underneath PED) and Bruch’s membrane depression under serosanguinous PED were found to have the highest value.

## Discussion

PCV is an exudative maculopathy with features similar to wet AMD. Identical characteristics in appearance features of AMD and PCV make it hard to differentiate them without advanced diagnostic examination. It is projected that the proportion of blindness attributable to AMD increases to 288 million affected persons in 2040 [2, 7]. As Asia currently accounts for 60% of the world’s population, this will eventually contribute to the highest global prevalence [2, 3]. Due to fact that AMD is the third leading cause of blindness in East Asia, it is crucial to differentiate the diagnosis of PCV and AMD since they have different approaches in treatment. PCV has to be taken in the context of its prevalence found in patients with wet AMD. It was shown that 22.3–61.6% of Asians who present with presumed wet AMD actually have PCV [2, 7].

Differentiation between PCV and wet AMD cannot be made merely on eye examination. As such, imaging modality is crucial to make sharp diagnoses and disease evaluations over time. While ICGA remains the gold standard, this study has demonstrated that OCT is a useful and informative tool in diagnosis of PCV. It provides qualitative and quantitative measurement, quick procedure, lower cost, and non-invasive imaging.

Most of the reported studies were hospital-based, and the paucity of PCV prevalence alone made it was hard to count the real predictive value of OCT in the population. The Beijing Eye Study 2011 attempted to estimate the prevalence of PCV using clinical findings and OCT (double-layer sign and high dome-shaped PED) [3]. In this study, they found PCV prevalence of 0.3% ± 0.1% (0.1–0.4) [2, 7]. As they did not use ICGA to confirm the findings, thus the result should be regarded to be presumptive rather than a definitive case. Additionally, the OCT biomarkers were limited and not specific for PCV. Therefore, it is best to expect a larger number and anticipate based on data from wet AMD.

In this study, the real positive predictive value (PPV) and negative predictive value (NPV) could not be obtained as they are dependent on prevalence. Other indicators, such as sensitivity, specificity, AUC, DOR and SROC, revealed very good value in spite of different thresholds. Notwithstanding that each study described similar OCT biomarkers of PCV, formulating final recommendations for diagnostic criteria remained elusive. First, each study did not provide the same parameter in determining the value of the biomarker. Furthermore, the positive threshold of PCV in some studies was determined by expert’s opinions which may lead to bias within a study. There should be a multicenter study that analyzes how strong is each biomarker indicating the disease. Aside from it, a study of PCV prevalence can be conducted once diagnostic criteria by OCT is established.

Two studies aimed to make diagnostic criteria based on the highest sensitivity, specificity and AUC by given biomarkers and clinical appearance. Four major criteria were proposed by Chaikitmongkol et al. [18]: notched or hemorrhagic PED detected by fundus examination; sharply peaked PED; notched or multilobulated PED; and the hyperreflective ring surrounding hyporeflectivity detected using OCT. Identifying at least 2 of these 4 major criteria had high specificity (95%), sensitivity (95%), AUC (93%). Yang et al. [24] recommended at least 2 of 5 major criteria: subretinal orange nodule on fundus examination; sharply peaked PED; notched PED; bubble sign; and Bruch’s membrane depression under serosanguinous PED on OCT. The diagnostic strategy of using at least 2 of 5 major criteria gave the highest predictive accuracy of 0.90, 0.88 sensitivity, and 0.92 specificities.

Pachychoroid is a relatively novel concept of phenotype characterized by abnormal thick choroid [5, 29]. In 2013, Freund and colleagues discussed pachychoroid pigment epitheliopathy, and the discussion about it has continued to develop ever since [29]. Choroid thickness is affected by age, refraction status, axial length and many more. Many studies reported the normal subfoveal choroid thickness to be between 220 and 350 nm [29]. Pachychoroid is defined as the choroid thickness of 390 nm and higher [29]. As the understanding of PCV pathophysiology has evolved, some studies considered it as the spectrum of this disease. Choroidal thickness as the sign of PCV was brought up by Chang et al. [4] and Yang et al. [24] Different results surfaced between these studies, in which Yang et al. [24] found that pachychoroid did not add more value in assessing PCV. Apart from that, Chang et al. [4] found it to be significant. The root of this difference could lie in the parameter thickness set by each study, as Chang et al.[4] present it lower than the common pachychoroid definition.

The limitation of this study included a small number of studies, where each study was performed in limited population variants such as Thai, Korean and Chinese ethnicity, and this study evaluated only the treatment-naïve patients; therefore, it is uncertain how OCT is able to detect PCV in patients whom already received treatment. However, if OCT is used to diagnose treatment-naïve PCV in the first visit, it is thought to be useful for treatment follow up.

This study will be suitable for a center in which ICGA is not available. ICGA may still be required in the settings where photodynamic therapy (PDT) with or without anti-VEGF is planned as in EVEREST Study [30]. Forasmuch as OCT is intended to diagnose the disease, the treatment protocol used in the PLANET (Aflibercept in Polypoidal Choroidal Vasculopathy) study can be applied. PLANET study showed that improvement of visual and functional outcomes could be achieved for most of the participants using Aflibercept as monotherapy [31].

Other imaging technologies (e.g., OCT angiography, en face OCT, SS-OCT) were not reported in this study. The principle of en face OCT is to reconstruct the dense volume of cross-sectional B scan data and project it onto a coronal or en face plane [32, 33]. This imaging technique will give assess and evaluate the interrelationship of hyper and hyporeflective oct lesions at a given depth segmentation. The ability of en face OCT to picture individual retinal layers on a transverse plane makes it beneficial, especially in diseases that affect certain retina layers. However, studies revealed that additional en face OCT did not help improve the predictive features of PCV [33].

In the emergence of multimodal imaging, it is thought that the use of more than one diagnostic imaging will help the clinician to understand more about the underlying pathogenesis, disease progression and treatment response [13]. It is yet to see how multimodal imaging will give value in diagnosing PCV. Unfortunately, this method cannot be easily implemented due to cost or health insurance issues. Multimodal imaging may have a greater impact on clinicians for learning purposes compared to patient’s necessities. Therefore, the use of OCT, especially for PCV, is requisite where ICGA is not available or when multimodal imaging is not preferable.

## Conclusion

OCT imaging has become widespread in ophthalmology because of its ability to visualize ocular cross-sectional structure at high resolution as a non-invasive and quick procedure. The sensitivity, specificity, SROC, and LR in this study indicate that OCT has a diagnostic value to establish PCV diagnosis. Compared to ICGA as the gold standard diagnostic tool for visualizing the PCV, OCT is more widely available. Nevertheless, deciding the diagnostic criteria is still problematic because each study did not use the same threshold despite the similar features. Acknowledging its ability to identify sharply peaked PED, notched PED, bubble sign as the most common features and multiple PED and double-layer sign as an additional marker, SD-OCT provides a high diagnostic value for PCV. Nevertheless, related to the limitations of studies that included only treatment- naïve patients, it is uncertain how OCT can detect PCV in patients who already received treatment. Therefore, further studies on the diagnosis of non-treatment naïve PCV and treatment response using OCT may be warranted.

## Data Availability

Not applicable.

## References

[1] Cheung CMG, Lai TYY, Teo K, Ophth M, Ruamviboonsuk P, Chen S (2020). Polypoidal choroidal vasculopathy consensus nomenclature and none indocyanine green angiograph diagnostic criteria from the Asia-Pacific Ocular Imaging Society PCV Workgroup. Ophthalmology.

[2] Wong CW, Wong TY, Ming C, Cheung G (2015). Polypoidal choroidal vasculopathy in Asians. J Clin Med.

[3] Li Y, You QS, Wei WB, Xu J, Cheng CX, Wang YX (2011). Polypoidal choroidal vasculopathy in adult Chinese: the Beijing eye study. Ophthalmology.

[4] Chang YS, Kim JH, Kim JW, Lee TG, Kim CG (2016). Optical coherence tomography-based diagnosis of polypoidal choroidal vasculopathy in Korean patients. Korean J Ophthalmol.

[5] Ming C, Cheung G, Lai TYY, Ruamviboonsuk P, Chen S, Chen Y (2018). Polypoidal choroidal vasculopathy definition, pathogenesis, diagnosis, and management. Ophthalmology.

[6] Nakashizuka H, Mitsumata M, Okisaka S, Shimada H (2008). Clinicopathologic findings in polypoidal choroidal vasculopathy. IOVS.

[7] Chaikitmongkol V, Cheung CMG, Koizumi H, Govindahar V, Chhablani J, Lai TYY (2020). Latest developments in polypoidal choroidal vasculopathy : Epidemiology, etiology, diagnosis, and treatment. Asia-Pacific J Ophthalmol.

[8] Lim TH, Laude A, Tan CSH (2010). Polypoidal choroidal vasculopathy : an angiographic discussion. Eye.

[9] Tan CSH, Ngo WK, Lim LW, Lim TH (2014). A novel classification of the vascular patterns of polypoidal choroidal vasculopathy and its relation to clinical outcomes. Br J Ophthalmol.

[10] Chaikitmongkol V, Khunsongkiet P, Patikulsila D, Ratanasukon M, Watanachai N, Jumroendararasame C (2018). Color fundus photography, optical coherence tomography, and fluorescein angiography in diagnosing polypoidal choroidal vasculopathy. Am J Ophthalmol.

[11] Kumar S, Nakashizuka H, Jones A, Lambert A, Zhao X, Shen M (2017). Proteolytic degradation and inflammation play critical roles in polypoidal choroidal vasculopathy. Am J Pathol.

[12] Imamura Y, Engelbert M, Iida T, Freund KB, Yannuzzi LA (2010). Polypoidal choroidal vasculopathy: a review. Surv Ophthalmol.

[13] Zhang J, Yu Z, Liu L (2015). Multimodality imaging in diagnosing polypoidal choroidal vasculopahty. Optom Vis Sci.

[14] Tan CS, Ngo WK, Chen JP, Tan NW, Lim TH (2015). EVEREST study report 2: imaging and grading protocol, and baseline characteristics of a randomised controlled trial of polypoidal choroidal vasculopathy. Br J.

[15] Tan CS, Ngo WK, Lim LW, Tan NW, Lim TH, Tan CS (2016). EVEREST study report 3: diagnostic challenges of polypoidal choroidal vasculopathy. Lessons learnt from screening failures in the EVEREST study. Graefe’s Arch Clin Exp Ophthalmol..

[16] Meira J, Marques ML, Falcão-reis F, Gomes ER, Carneiro Â (2020). Immediate reactions to fluorescein and indocyanine green in retinal angiography: review of literature and proposal for patient’s evaluation. Clin Ophthalmol.

[17] Yi Z, Jing YAO, Xiaohua W, Lin Z, Lijun W, Jianming W (2017). Sensitivity and specificity of optical coherence tomography in diagnosing polypoidal choroidal vasculopathy. J South Med Universiity.

[18] Chaikitmongkol V, Kong J, Khunsongkiet P, Patikulsila D, Sachdeva M, Chavengsaksongkram P (2019). Sensitivity and specificity of potential diagnostic features detected using fundus photography, optical coherence tomography, and fluorescein angiography for polypoidal choroidal vasculopathy. JAMA Ophthalmol.

[19] Teo KYC, Ophth M, Cheung GCM (2019). New concepts in polypoidal choroidal vasculopathy imaging: a focus on optical coherence tomography and optical coherence tomography angiography. Asia Pac J Ophthalmol.

[20] DE Salvo G, Vaz-pereira S, Keane PA, Tufail A, Liew G (2014). Sensitivity and specificity of spectral-domain optical coherence tomography in detecting idiopathic polypoidal choroidal vasculopathy. Am J Ophthalmol.

[21] Gabriele ML, Wollstein G, Ishikawa H, Kagemann L, Xu J, Folio LS (2011). Optical coherence tomography: history, current Status, and laboratory Work. IOVS.

[22] Fujimoto J, Swanson E (2016). The development, commercialization, and impact of optical coherence tomography. IOVS.

[23] Kim JH, Kang SW, Kim T, Kim SJ, Ahn J (2013). Structure of polypoidal choroidal vasculopathy studied by colocalization between tomographic and angiographic lesions. Am J Ophthalmol.

[24] Yang J, Yuan M, Wang E, Xia S, Chen Y (2019). Noninvasive multimodal imaging in diagnosing polypoidal choroidal vasculopathy. BMC Ophthalmol.

[25] Hamzah F, Shinojima A, Mori R, Yuzawa M (2014). Choroidal thickness measurement by enhanced depth imaging and swept-source optical coherence tomography in central serous chorioretinopathy. BMC Ophthalmol.

[26] Song G, Chu KK, Kim S, Crose M, Cox B, Evan T (2019). First clinical application of low-cost OCT. Transl VIs Scie Technol.

[27] Kempen JH, Sugar EA, Jaffe GJ, Acharya NR, Dunn JP, Elner SG (2013). Fluorescein angiography versus optical coherence tomography for diagnosis of uveitic cacular edema. Ophthalmology.

[28] Liu R, Li J, Li Z, Yu S, Yang YU, Yan H (2016). Distinguishing polypoidal choroidal vasculopathy from typical neovascular age-related macular degeneration based on spectral domain optical coherence tomography. Retina.

[29] Akkaya S (2017). Spectrum of pachychoroid diseases. Int Ophthalmol.

[30] Tan CS, Lim LW, Ngo WK, Lim TH, EVEREST Study Group. (2018). EVEREST Report 5: Clinical outcomes and treatment response of polypoidal choroidal vasculopathy subtypes in a multicenter, randomized controlled trial. Retina.

[31] Lee WK, Lida T, Ogura Y, Chen S-J, Wong TY, Mitchell P (2018). Efficacy and safety of intravitreal aflibercept for polypoidal choroidal vasculopathy in the PLANET study a randomized clinical trial. JAMA Ophthalmol.

[32] Lian R, Lai JC, Wee R (2018). Sensitivity and specificity of detecting polypoidal choroidal vasculipathy with en face optical coherence tomography and optical coherence tomography angiography. Retina.

[33] Kokame GT, Shantha JG, Hirai K, Ayabe J (2016). En face spectral-domain optical coherence tomogrpahy for the diagnosis and evaluation of polypoidal choroidal vasculopathy. Ophthalmic Surg Lasers Imaging Retin.

